# Safety, efficacy, and drug survival of the infliximab biosimilar CT‐P13 in post‐marketing surveillance of Japanese patients with psoriasis

**DOI:** 10.1111/1346-8138.16508

**Published:** 2022-07-07

**Authors:** Akimichi Morita, Kiyohiro Nishikawa, Fumika Yamada, Keiichi Yamanaka, Hideki Nakajima, Mamitaro Ohtsuki

**Affiliations:** ^1^ Department of Geriatric and Environmental Dermatology Nagoya City University Graduate School of Medical Sciences Nagoya Japan; ^2^ Quality and Pharmacovigilance Division Pharmaceuticals Group, Nippon Kayaku Co., Ltd. Tokyo Japan; ^3^ Department of Dermatology Mie University Graduate School of Medicine Tsu Japan; ^4^ Department of Dermatology Kochi Medical School, Kochi University Nankoku Japan; ^5^ Department of Dermatology Jichi Medical University Shimotsuke Japan; ^6^ Asajes Ventures Tokyo Japan

**Keywords:** biosimilar, CT‐P13, infliximab, post‐marketing surveillance, psoriasis

## Abstract

Based on extrapolation of similar clinical outcomes in rheumatoid arthritis to the originator infliximab (IFX) in randomized clinical trials, the first biosimilar antibody CT‐P13 was approved for the treatment of psoriasis. To evaluate the safety, efficacy, and drug survival of CT‐P13 for psoriasis in real‐world clinical practice, prospective post‐marketing surveillance was conducted in 165 Japanese psoriasis patients. During a 1‐year follow‐up period, adverse drug reactions (ADRs) occurred in 29 patients (17.6%). Infusion reaction was the most frequent ADR (6.7%), and mild pneumonia was reported as the only case of infection. Serious ADRs were reported in two patients (1.2%): acute cholecystitis and interstitial pneumonia. The interstitial pneumonia developed after a single infusion of CT‐P13 and the patient died of respiratory failure. In naive patients to biologic therapy (*n* = 44), the Psoriasis Area Severity Index (PASI) decreased rapidly after the start of CT‐P13 treatment, and response rate achieving an absolute PASI score <1 was 55% at 30 weeks. The response rate was high (78%) in patients with psoriatic arthritis, and 40% and 20% in those in plaque psoriasis and pustular psoriasis, respectively. Of patients switched from IFX to CT‐P13 mainly for nonmedical reasons (*n* = 105), 57% had already reached PASI <1 by pretreatment with IFX and CT‐P13 maintained this status. The incidence of ADRs in this patient group was low and the drug survival rate was as high as 74%, even at 1 year, which was significantly higher than that in the naïve patient group (47%). Patients switched from other biologics for medical reasons (*n* = 16) responded similarly to biologic‐naïve patients, but drug survival was lower (24%). In conclusion, CT‐P13 showed excellent effectiveness as a first‐line therapy, no clinical difficulties in switching from IFX, and usefulness in patients who failed other biologics. CT‐P13 could be a cost‐effective alternative to IFX for the treatment of psoriasis.

## 1 INTRODUCTION

Psoriasis is a chronic inflammatory dermatologic disease mediated by cytokines, including tumor necrosis factor‐α (TNFα), interleukin‐17, and interleukin‐23. In randomized clinical trials, biologics targeting these cytokines have shown therapeutic efficacy as an induction therapy for moderate‐to‐severe psoriasis.^1^ Clinical management of psoriasis and psoriatic arthritis has been transformed by the introduction of biologics, starting with infliximab (IFX), an anti‐TNFα antibody.

CT‐P13, the first approved IFX biosimilar, was developed to reduce the economic burden on healthcare and improve patient access to treatment. In randomized controlled clinical studies comparing IFX and CT‐P13 in patients with rheumatoid arthritis and ankylosing spondylitis, the pharmacokinetics, safety profile, and efficacy of CT‐P13 were comparable to those of IFX.^2^, ^3^, ^4^, ^5^ CT‐P13 was approved for the treatment of various inflammatory diseases, including inflammatory bowel disease and psoriasis, by extrapolation without undergoing clinical trials. After its approval, two randomized controlled studies and several observational clinical studies demonstrated a similar safety profile, immunogenicity, and comparable efficacy to the originator IFX.^6^, ^7^, ^8^, ^9^ However, studies of CT‐P13 in psoriasis patients are few and include only a limited number of patients.^6^, ^10^, ^11^

In 2014, we initiated a series of prospective post‐marketing surveillance (PMS) studies of CT‐P13 in a variety of Japanese patients and reported the interim results of the PMS in rheumatoid arthritis and inflammatory bowel disease.^12^, ^13^, ^14^ Here we present the PMS results regarding the safety, efficacy, and drug survival of CT‐P13 in 165 Japanese patients with psoriasis. In this report, we first analyzed the clinical effects of CT‐P13 in biologic‐naïve patients with four types of psoriasis and compared them with previous PMS data of the originator IFX.^15^ Second, we examined whether switching to CT‐P13 from IFX is acceptable in terms of safety and maintaining clinical effectiveness. Third, we explored the clinical effects of CT‐P13 in patients who switched from previous treatment with other biologics due to an inadequate response or adverse events (AEs). In addition, we identified clinical risk factors for adverse drug reactions (ADRs) and predictors for the efficacy of CT‐P13 by multivariable logistic regression analyses.

## 2 METHODS

### 2.1 Post‐marketing surveillance

Nippon Kayaku Co., Ltd initiated PMS in patients with psoriasis after the approval of CT‐P13 in Japan. Patients were enrolled prospectively from July 2014 to December 2019 and were followed up for 1 year after the start of CT‐P13 therapy. To minimize patient selection bias, we used a centralized system for registering all patients at the contracted clinical sites. Patients were defined as those with psoriasis unsuccessfully treated with conventional therapies and who received treatment with CT‐P13 according to the Japanese Dermatological Association's 2013 guidance for use of biologics for psoriasis.^16^ The CT‐P13 regimen was recorded and changes in the scheduled dosage and dosing interval were reported along with the reasons for those changes. Case report forms were collected from each patient at 6 months and 1 year after the start of treatment.

The PMS was required by the Ministry of Health, Labour and Welfare (MHLW) as a condition for the approval of CT‐P13. The protocol and ethical considerations of the PMS study (Code IFX22) were assessed by internal review board members and approved by the MHLW, and no additional formal ethics committee approval was needed. The PMS was conducted in accordance with the Good Post‐marketing Study Practice Ordinance of the MHLW and informed consent from individual patients was not required.

### 2.2 Safety

All AEs, including subjective/objective findings and laboratory test data, were collected and the causal relationship of AEs to CT‐P13 was evaluated. AEs and ADRs for which a causal relationship with CT‐P13 was not ruled out were coded in accordance with the System Organ Class and Preferred Term listed in the Medical Dictionary for Regulatory Activities (MedDRA/J; version 24.0). ADRs of particular interest were infusion reaction, serious infections (including tuberculosis), interstitial lung disease, and malignant neoplasms.

### 2.3 Efficacy

The efficacy of CT‐P13 for psoriasis was evaluated by the absolute Psoriasis Area Severity Index (PASI) (graded 0, <1, <2, <5, ≥5), as well as the PASI response to achieve a reduction in the baseline PASI (PASI 100, 90, 75, 50).^17^ Physician's Global Assessment (PGA) scores of 0 or 1 (clear or almost clear) and a Dermatology Life Quality Index (DLQI) <1 were also considered to indicate effectiveness.^18^, ^19^ Effects on joint symptoms in psoriatic arthritis patients were also evaluated according to the Disease Activity Score in 28 joints with C‐reactive protein (DAS28‐CRP) with cut‐off values for high, moderate, and low disease activity of 5.1, 3.2, and 2.6, respectively. The European League Against Rheumatism (EULAR) response criteria, with cut‐off decreases in DAS28 of 0.6 and 1.2, were also applied for evaluation.^20^, ^21^

The data obtained on the nearest day to the scheduled administration time points (weeks 2, 6, and every 8 weeks thereafter for IFX‐naïve patients, and every 8 weeks for patients switched from IFX to CT‐P13) were used as the representative values.

### 2.4 Statistical analysis

The drug survival of CT‐P13 was plotted using the Kaplan–Meier method, with the last administration as an event. Patients who discontinued treatment with CT‐P13 after a single dosing were excluded from the plots. Differences between patient groups were analyzed using the log‐rank test.

Univariate analysis followed by multivariable analysis was performed using a logistic regression model to explore risk factors for the occurrence of infusion reactions and other ADRs. The logistic regression model was also applied for efficacy analysis to find predictors for a drug response (absolute PASI <1) between 14/16 and 30/32 weeks after CT‐P13 administration. As explanatory clinical variables, 10 patient background factors (sex, age, type of psoriasis, disease duration, disease severity, body weight, body mass index [BMI], smoking history, history of drug allergy, and comorbidities) and three therapeutic factors (prior biologics, concomitant use of methotrexate [MTX], concomitant topical use of steroids or vitamin D) were used. Sex, age, and factors with *p* < 0.2 in the univariate analysis were incorporated into the multivariable logistic model, and significant clinical factors were selected by the stepwise method. The odds ratio (OR) and the two‐sided Wald 95% confidence interval (95% CI) were estimated for each covariate.

## 3 RESULTS

### 3.1 Patient characteristics

In this prospective PMS of CT‐P13, 168 patients were registered, but case report forms for three patients were not available. The 165 patients who received at least one infusion of CT‐P13 were included in the safety analysis set and had been followed up for 1 year. Evaluable efficacy data were missing from eight patients, and 157 patients were included in the efficacy analysis set (Figure S1).

Representative patient characteristics, disease status, prior therapy, and concomitant medications are summarized in Table 1. Fifty‐five patients had plaque psoriasis (33%), 104 had psoriatic arthritis (63%), 20 had pustular psoriasis (12%), and two had psoriatic erythroderma (1%). Sixteen patients (10%) had multiple types of psoriasis: plaque psoriasis with psoriatic arthritis (*n* = 10), psoriatic arthritis with pustular psoriasis (*n* = 3), plaque psoriasis with pustular psoriasis (*n* = 2), and pustular psoriasis with psoriatic erythroderma (*n* = 1). According to their use of biologics prior to PMS entry, the patients were classified into three groups: (1) 44 patients (27%) who were naïve to biologics and treated with CT‐P13 as the first biologic therapy; (2) 105 patients (64%) who had been treated with the originator IFX and switched to CT‐P13 for mainly nonmedical reasons such as reduction of drug cost burden and hospital policy; and (3) 16 patients (10%) who had received other biologics and switched to CT‐P13 for medical reasons such as AEs and inadequate response.

**TABLE 1:** Baseline characteristics of the 165 patients in the safety analysis set

	Plaque psoriasis	Psoriatic arthritis	Pustular psoriasis	Psoriatic erythroderma	Naïve to biologics	Switched from IFX	Switched from other biologics
	*n* = 55	*n* = 104	*n* = 20	*n* = 2	*n* = 44	*n* = 105	*n* = 16
Patient characteristics							
Sex (male rate)	73%	69%	50%	100%	61%	73%	69%
Age (years)	53.2 ± 11.7	53.9 ± 13.3	51.6 ± 12.3	51, 63	52.8 ± 15.2	53.6 ± 11.4	57.9 ± 15.1
Disease duration (years)	17.9 ± 11.5	14.9 ± 10.7	19.7 ± 10.9	0.2, 11.0	11.7 ± 9.1	18.5 ± 11.4	11.2 ± 8.3
BMI (kg/m^2^)	25.4 ± 5.5	25.1 ± 4.8	25.9 ± 5.6	21.1, 21.1	25.1 ± 4.7	24.8 ± 4.8	28 ± 5.8
Presence of comorbidities	40%	47%	75%	50%	57%	46%	44%
Disease status							
PASI score	5.4 ± 9.5	3.4 ± 5.5	6.5 ± 13.5	15.2, 54	11.6 ± 10.9	1.9 ± 6.0	5.8 ± 5.5
BSA (%)	10.7 ± 18.2	5.8 ± 11.9	20.6 ± 34.2	25	27.2 ± 27.6	3.2 ± 8.9	9.5 ± 13.6
PGA	1.4 ± 1.5	1.3 ± 1.4	1.7 ± 1.7	4	3.1 ± 1.3	0.7 ± 0.9	1.9 ± 1.3
DLQI	6.2 ± 7.7	5.3 ± 7.1	6.5 ± 10.2	11, 12	9.8 ± 9.8	2.1 ± 3.2	10.4 ± 6.4
NAPSI	1.2 ± 1.9	1.0 ± 1.8	0.9 ± 1.8	4	2.5 ± 2.3	0.7 ± 1.4	0.8 ± 1.7
DAS28‐CRP	–	2.4 ± 1.2	–	–	–	–	–
Severe disease^a^	27%	18%	33%	100%	66%	8%	36%
Prior therapy							
Prior biologics							
None	18%	30%	30%	50%	100%	–	–
1 drug	71%	58%	70%	50%	–	88%	63%
2+ drugs	11%	13%	0%	0%	–	12%	38%
Prior medication							
Cyclosporine	31%	25%	45%	50%	20%	32%	25%
Topical steroid	82%	59%	90%	100%	68%	74%	38%
Topical vitamin D	67%	48%	60%	100%	55%	58%	31%
Phototherapy	24%	13%	40%	0%	9%	24%	13%
Surgery	7%	18%	30%	0%	16%	15%	31%
Medication							
Dose of CT‐P13 (mg/kg)	5.8 ± 1.5	5.6 ± 1.5	5.6 ± 1.2	5.4, 6.3	5.2 ± 1.0	5.9 ± 1.5	5.8 ± 1.8
Combination							
MTX	25%	49%	20%	50%	36%	38%	50%
Topical steroid	62%	42%	80%	100%	68%	52%	25%
Topical vitamin D	56%	36%	50%	100%	52%	45%	13%
Phototherapy	4%	2%	0%	0%	0%	4%	0%

*Note:* Values are expressed as % or mean ± standard deviation.

Abbreviations: BMI, body mass index; BSA, body surface area; DAS28‐CRP, disease activity score in 28 joints with C‐reactive protein; DLQI, Dermatology Life Quality Index; IFX, infliximab; MTX, methotrexate; NAPSI, Nail Psoriasis Severity Index; PASI, Psoriasis Area and Severity Index; PGA, Physician Global Assessment.

^a^ Severe disease is defined by the rule of 10s (PASI >10, BSA >10%, or DLQI >10).

Approximately 70% of the patients in each group were male, and the average age of each group was similar (mid‐50s). Patients with psoriatic arthritis had a shorter disease duration and lower disease status. For psoriatic arthritis, prior treatment with cyclosporine, topical steroids and vitamin D, and phototherapy was less frequent. Concomitant use of topical steroids, vitamin D, and phototherapy was also infrequent in psoriatic arthritis patients, although MTX was combined in 49% of patients in this group. In patients with pustular psoriasis, the disease duration was longer, comorbidities were commonly observed (75%), and the disease status was severe.

In the naïve patient group, the average PASI, body surface area (BSA), and DLQI were 11.6, 27.2%, and 9.8, respectively, and 66% were considered to have severe psoriasis according to the rule of 10s (PASI >10, BSA >10% or DLQI >10).^22^ In contrast, in the group of patients who switched from IFX, all disease scores were low and only 8% of patients had severe psoriasis. Most of those patients (88%) had been treated with IFX alone as the first biologic therapy. The dose of CT‐P13 was almost the same as the last dose of IFX in most patients, but was increased to the higher from the standard dose of IFX in four patients and reduced to the standard dose of IFX dose in three patients. The disease status of patients who switched from other biologics was less severe than that of naïve patients, but 36% of patients had severe disease. Topical steroids or vitamin D were infrequently prescribed for these patients in pretreatment as well as in combination with CT‐P13. Approximately one‐third (38%) of the patients experienced treatment failure with at least two previous biologics before switching to CT‐P13.

### 3.2 Incidence of adverse drug reactions

Of the 165 patients in the safety analysis set, 44 AEs (26.7%) and eight serious AEs (4.8%) were reported during the 1‐year follow‐up period (Table 2). Among them, ADRs and serious ADRs were observed in 29 (17.6%) and two patients (1.2%), respectively. The most common ADR was infusion reaction (*n* = 11 patients, 6.7%), and although there were no serious cases, five patients discontinued treatment. In the infection category, only one case of pneumonia was reported. The patient developed pyrexia after seven doses of CT‐P13, but the symptoms were mild and resolved with antibiotics, and administration of CT‐P 13 was continued after a 4‐week delay. Tuberculosis did not occur. Examination for tuberculosis (tuberculin skin test, interferon gamma release assay, chest radiograph, and computed tomography) before CT‐P13 administration was performed in all but one patient. Interstitial pneumonia occurred in three patients. Two patients recovered after discontinuing CT‐P 13 without treatment, but one naive patient with psoriatic arthritis developed serious interstitial pneumonia 12 days after the first infusion of CT‐P13 following oral MTX at 8 mg/week for 12 days. Despite repeated pulse therapy with steroids and cyclophosphamide, the patient died of respiratory failure 45 days after administration. He was 59 years old with a 45‐year history of smoking, but no abnormalities were found on previous chest radiography and computed tomography. He had no comorbidities or past medical history, and no disorder was recorded in the medical interview. The causal relationship to CT‐P13 and/or MTX was reported to be unknown. The other serious ADR was acute cholecystitis. After the fifth dose of CT‐P13, the patient was hospitalized with fever and arthralgia. Abdominal computed tomography showed swelling of the gallbladder and gallstones in the gallbladder neck, and the patient recovered following antibiotic administration. Neoplasms were found in three patients during the observation period, but all were considered unrelated to CT‐P13. All reported AEs and ADRs classified by the System Organ Class are listed in Supporting Information Table S1.

**TABLE 2:** Incidence of AEs and ADRs to CT‐P13

	Incidence of AEs				Incidence of ADRs			
	Any AEs		Serious AEs		Any ADRs		Serious ADRs	
Total AEs/ADRs (*n* = 165)	44	(26.7%)	8	(4.8%)	29	(17.6%)	2	(1.2%)
Disease type								
Plaque psoriasis (*n* = 55)	16	(29.1%)	2	(3.6%)	11	(20.0%)	0	(0.0%)
Psoriatic arthritis (*n* = 104)	25	(24.0%)	5	(4.8%)	14	(13.5%)	1	(1.0%)
Pustular psoriasis (*n* = 20)	5	(25.0%)	1	(5.0%)	5	(25.0%)	1	(5.0%)
Psoriatic erythroderma (*n* = 2)	0	(0.0%)	0	(0.0%)	0	(0.0%)	0	(0.0%)
Prior therapy								
Naïve to biologics (*n* = 44)	15	(34.1%)	3	(6.8%)	14	(31.8%)	2	(4.5%)
Switched from IFX (*n* = 105)	24	(22.9%)	3	(2.9%)	12	(11.4%)	0	(0.0%)
Switched from other biologics (*n* = 16)	5	(31.3%)	2	(12.5%)	3	(18.8%)	0	(0.0%)
Category of AEs/ADRs by SOC (occurred in ≥5 patients)								
General disorders and administration site conditions	5	(3.0%)	0	(0.0%)	3	(1.8%)	0	(0.0%)
Hepatobiliary disorders	5	(3.0%)	2	(1.2%)	4	(2.4%)	1	(0.6%)
Infections and infestations	5	(3.0%)	1	(0.6%)	1	(0.6%)	0	(0.0%)
Injury, poisoning, and procedural complications	12	(7.3%)	0	(0.0%)	11	(6.7%)	0	(0.0%)
Investigations	5	(3.0%)	0	(0.0%)	5	(3.0%)	0	(0.0%)
Nervous system disorders	5	(3.0%)	0	(0.0%)	1	(0.6%)	0	(0.0%)
Respiratory, thoracic, and mediastinal disorders	9	(5.5%)	2	(1.2%)	6	(3.6%)	1	(0.6%)
Skin and subcutaneous tissue disorders	6	(3.6%)	0	(0.0%)	4	(2.4%)	0	(0.0%)
AEs/ADRs of particular interest								
Infusion reaction	11	(6.7%)	0	(0.0%)	11	(6.7%)	0	(0.0%)
Tuberculosis	0	(0.0%)	0	(0.0%)	0	(0.0%)	0	(0.0%)
Pneumonia	1	(0.6%)	0	(0.0%)	1	(0.6%)	0	(0.0%)
Interstitial lung disease	4	(2.4%)	1	(0.6%)	3	(1.8%)	1	(0.6%)
Neoplasm benign, malignant and unspecified^a^	3	(1.8%)	2	(1.2%)	0	(0.0%)	0	(0.0%)

*Note:* Data are expressed as patient number with AE/ADR (%).

Abbreviations: ADR, adverse drug reaction; AE, adverse event; IFX, infliximab; SOC, system organ class.

^a^ Squamous cell carcinoma of skin, metastasis to lymph nodes of breast cancer, and lymphoproliferative disorder.

Among psoriasis types, the incidence of ADRs in psoriatic arthritis patients was low (13.5%). While the incidence of ADRs in biologic‐naïve patients was 31.8%, that in patients who switched from IFX was 11.4%.

### 3.3 Risk factors for ADRs

Univariate and multivariable logistic regression analyses were performed for infusion reactions and other systemic ADRs separately, as the characteristics and onset time differed (Supporting Information Table S2 and Table 3). In the multivariable analysis, the incidence of an infusion reaction was significantly lower in patients who switched from IFX than in naïve patients (OR = 0.43, 95% CI 0.08–2.42, *p* = 0.012) and significantly higher in patients switched from other biologics than in naïve patients (OR = 19.7, 95% CI 1.33–291, *p* = 0.009). Comorbidities were associated with infusion reactions (OR = 41.6, 95% CI 1.87–923, *p* = 0.018). Combination treatment with MTX tended to suppress the occurrence of an infusion reaction (OR = 0.14, 95% CI 0.01–1.40, *p* = 0.093). In addition, in the univariate analysis, the incidence of an infusion reaction in patients with severe disease and a history of drug allergy was significantly higher, although no significant associations were detected in the multivariable analysis.

**TABLE 3:** Multivariable logistic regression analysis of baseline clinical factors associated with incidence of infusion reactions and other ADRs

Baseline factor	Category (reference)	Infusion reactions			Other ADRs		
		OR (95% CI)		*p* value	OR (95% CI)		*p* value
Type of psoriasis	Plaque psoriasis (reference)						
	Psoriatic arthritis	0.30	(0.04–2.16)	0.112	0.19	(0.06–0.66)	0.007**
	Pustular psoriasis	1.54	(0.22–10.9)	0.243	0.96	(0.19–4.89)	0.300
Prior biologics (Patient group)	Naïve (reference)						
	Switched from IFX	0.43	(0.08–2.42)	0.012*	0.15	(0.05–0.50)	0.271
	Switched from other biologics	19.7	(1.33–291)	0.009**	0.11	(0.01–1.07)	0.253
Sex	Female (reference)						
	Male	0.81	(0.18–3.62)	0.782	5.16	(1.18–22.6)	0.029*
Comorbidities	No (reference)						
	Yes	41.6	(1.87–923)	0.018*	2.02	(0.66–6.13)	0.217
Concomitant use of MTX	No (reference)						
	Yes	0.14	(0.01–1.40)	0.093	3.70	(1.24–11.0)	0.019*

*p < 0.05, **p < 0.01.

Abbreviations: ADRs, adverse drug reactions; CI, confidence interval; IFX, infliximab; OR, odds ratio; MTX, methotrexate.

The incidence of other ADRs was significantly lower in patients with psoriatic arthritis (OR = 0.19, 95% CI 0.06–0.66, *p* = 0.007). Male sex and combination treatment with MTX were significant risk factors for ADRs (OR = 5.16, 95% CI 1.18–22.6, *p* = 0.029, and OR = 3.70, 95% CI 1.24–11.0, *p* = 0.019, respectively).

### 3.4 Efficacy by psoriasis type

**FIGURE 1:**
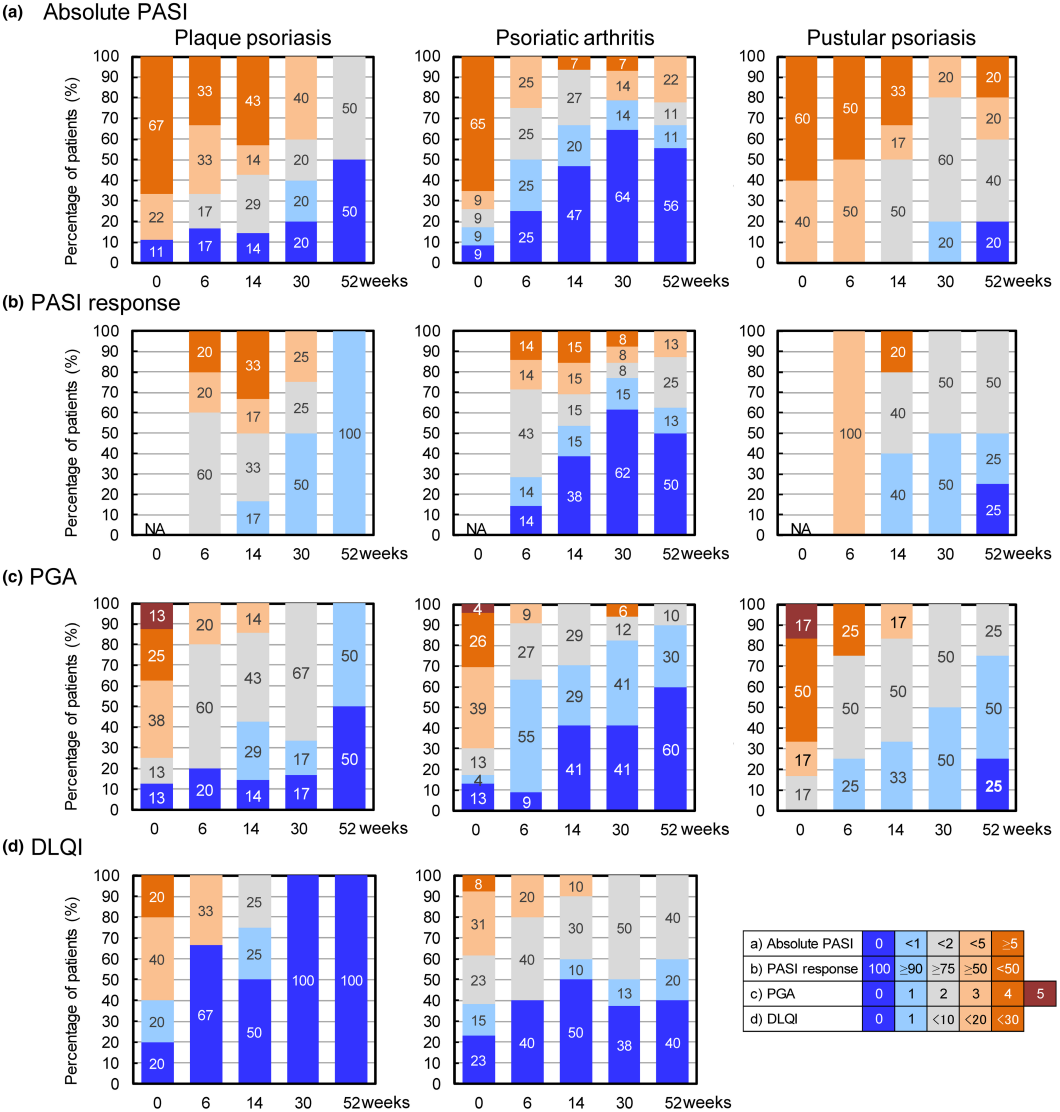
Efficacy of CT‐P13 in naïve patients with plaque psoriasis (*n* = 10), psoriatic arthritis (*n* = 31), and pustular psoriasis (*n* = 6). Changes in distribution of patients by (a) absolute PASI, (b) PASI response, (c) PGA, and (d) DLQI over time from baseline to week 52. DLQI in patients with pustular psoriasis was not presented due to the small number of patients. DLQI, Dermatology Life Quality Index; IFX, infliximab; N/A, not applicable; PASI, Psoriasis Area and Severity Index; PGA, Physician Global Assessment.

The efficacy of CT‐P13 for three psoriasis types was evaluated by changes in the PASI, PGA, and DLQI in biologic‐naive patients in the efficacy analysis set (Figure 1). In plaque psoriasis, the proportion of patients with an absolute PASI of 0 gradually increased after the initiation of CT‐P13 therapy. In psoriatic arthritis, the proportion of patients achieving an absolute PASI of 0 increased more rapidly than in those with plaque psoriasis and exceeded 60%. A smaller proportion of patients with pustular psoriasis achieved an absolute PASI of 0. Patients with PASI >5, which accounted for approximately 60% of patients in all psoriasis types at the start of CT‐P13, decreased with CT‐P13 treatment. The efficacy of CT‐P13 treatment was also confirmed by the PASI response, although the number of evaluable patients was reduced because the PASI response could not be calculated when the baseline PASI was 0. Especially in patients with psoriatic arthritis, PASI 100 was achieved in 62% of the patients at week 30. A similar objective response was observed with the PGA. The proportion of patients with PGA 0 or 1 increased with CT‐P13 treatment, and the PGA also indicated higher efficacy in psoriatic arthritis patients. On the other hand, with respect to the quality of life scored by the patients, the percentage of patients with DLQI <1 was higher in patients with plaque psoriasis than in patients with psoriatic arthritis. Objective evaluation by physicians and subjective patient‐reported outcomes were inconsistent between these types of psoriasis.

In these three types of psoriasis, CT‐P13 was evaluated to be markedly effective, but its efficacy for psoriatic erythroderma could not be clearly determined because there were only two patients. Nevertheless, one patient with psoriatic erythroderma showed a good response to CT‐P13, and both the PASI and DLQI reached 0 from 54 and 12 at baseline, respectively. In the other patient, the baseline PASI 15 and DLQI 11 did not decrease and CT‐P13 was switched to secukinumab after 4 weeks.

### 3.5 Efficacy by previous treatment status with biologics

**FIGURE 2:**
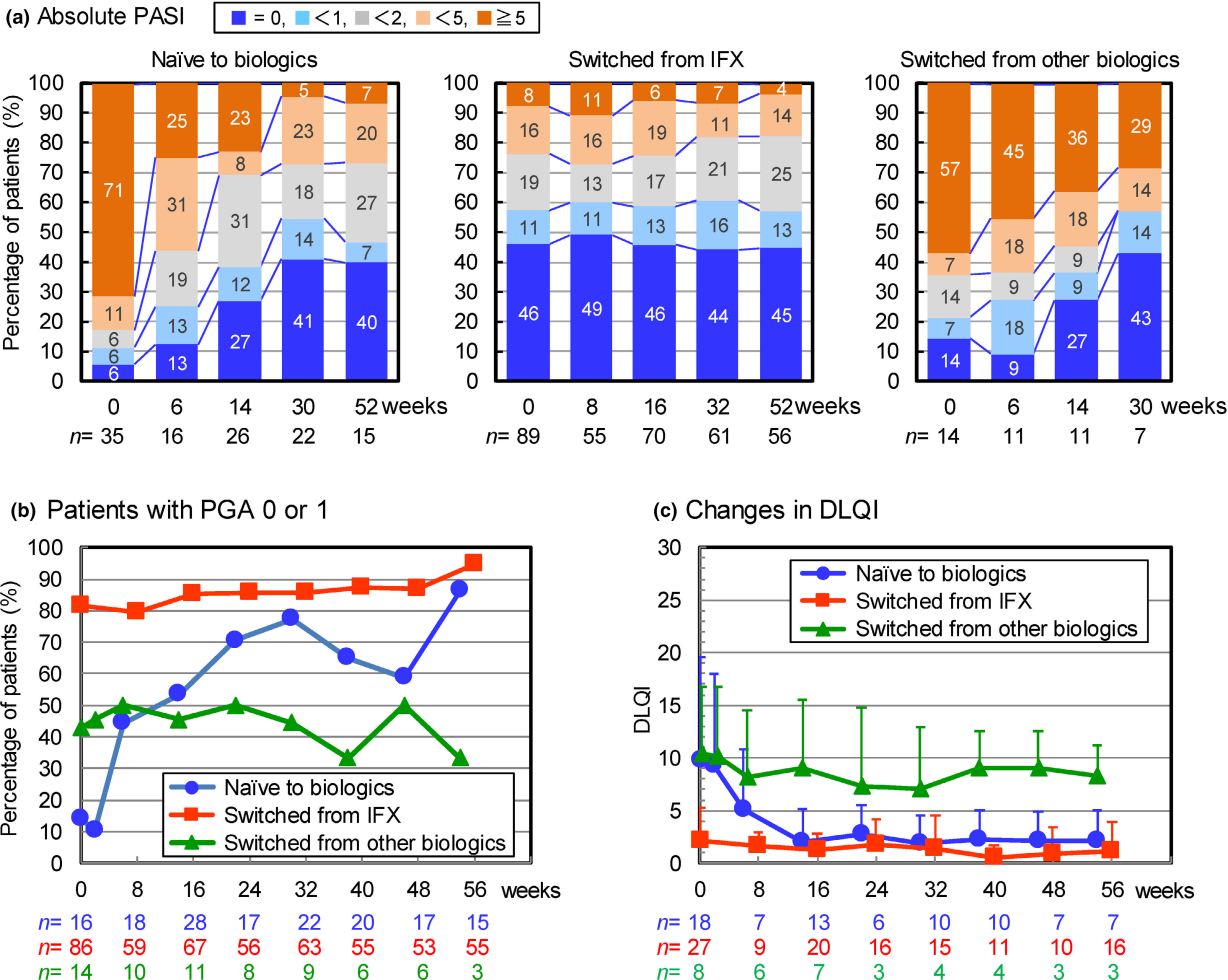
Efficacy of CT‐P13 in patients who were naïve to or switched from biologics. (a) Changes in distribution of patients by absolute PASI, (b) changes in proportion of patients with PGA score of 0 or 1, and (c) changes in DLQI (mean ± standard deviation) over time from baseline to week 56. DLQI, Dermatology Life Quality Index; IFX, infliximab; PASI, Psoriasis Area and Severity Index; PGA, Physician Global Assessment.

The efficacy of CT‐P13 was evaluated in three patient groups: naïve patients to biologics, patients switched from IFX, and patients switched from other biologics (Figure 2). At the start of treatment, 71% of naïve patients had an absolute PASI ≥5. The PASI score decreased rapidly after treatment with CT‐P13 and 55% of patients achieved an absolute PASI <1 at week 30. In patients who switched from IFX, 57% of patients already had an absolute PASI <1 by prior treatment with IFX and efficacy was maintained in approximately 60% of patients after switching to CT‐P13. In patients switched from other biologics, more than half achieved an absolute PASI <1 at week 30, but 29% of the patients continued to have an absolute PASI ≥5.

In naïve patients, the proportion of patients with a PGA score of 0 or 1 increased from 14% to 77% at week 30, and the DLQI decreased from 9.8 to 1.9 at week 30. In patients switched from IFX, 81% had PGA 0 or 1 at the start of treatment and the baseline DLQI was 2.1. Treatment with CT‐P13 maintained good patient status and further improved the efficacy indices. In patients switched from other biologics, changes in the PGA and DLQI from baseline were marginal.

### 3.6 Efficacy for joint symptoms in patients with psoriatic arthritis

In patients with psoriatic arthritis, the efficacy of CT‐P13 for joint symptoms was also evaluated based on the change in the DAS28‐CRP (Supporting Information Figure S2). The mean DAS28‐CRP of naïve patients decreased rapidly from 3.4 at baseline to 2.2 at week 14 and then gradually dropped to 1.4 at week 30. At week 14, 83% of naïve patients achieved remission (DAS28‐CRP <2.6) and 88% showed a good or moderate EULAR response. In contrast, the DAS28‐CRP of patients switched from IFX was already low (1.6) before starting CT‐P13, and remission was obtained in 90% of patients due to prior treatment with the originator IFX. The DAS28‐CRP in these patients decreased further to around 1, but the low baseline value limited the margin for producing a good EULAR response.

### 3.7 Efficacy by body region

Comparison of the efficacy of CT‐P13 in each body region showed that the highest PASI 100 response rate was in the head (53%) followed by the arms and trunk (40% and 41%, respectively). Efficacy in the legs was lowest, with only 30% of patients achieving PASI 100 in the legs (Supporting Information Figure S3).

### 3.8 Predictors for efficacy

**FIGURE 3:**
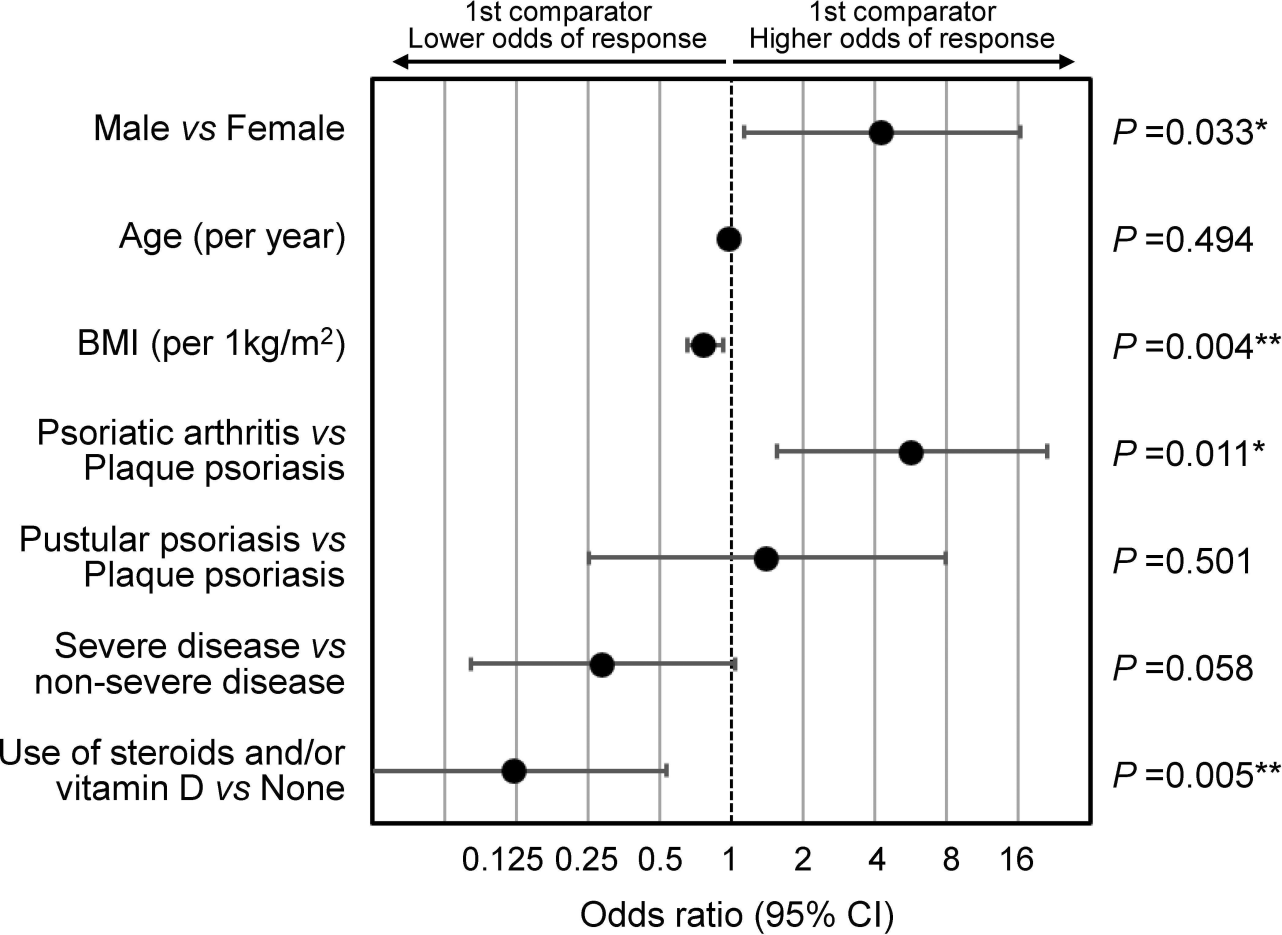
Multivariable logistic regression analysis of baseline clinical factors associated with response to CT‐P13, that is achieving an absolute PASI <1 between 14/16 and 30/32 weeks after CT‐P13 administration. *p < 0.05, **p < 0.01. BMI, body mass index; CI, confidence interval. PASI, Psoriasis Area and Severity Index.

We explored the association of clinical factors with the response to CT‐P13 by univariate and multivariable logistic regression analysis (Supporting Information Table S3 and Figure 3). The response rate for reaching an absolute PASI <1 was higher in male patients than in female patients (63% vs 53%) and male sex was a significant factor associated with a response in multivariable analysis (OR = 4.28, 95% CI 1.12–16.3, *p* = 0.033). An increased BMI was a negative predictor of response (OR = 0.77, 95% CI 0.65–0.92, *p* = 0.004) and the response rate steadily dropped with increasing the BMI and body weight (Supporting Information Figure S4). The presence of psoriatic arthritis was an independent predictor of a good response (OR = 5.73, 95% CI 1.54–21.2, *p* = 0.011). Association of a poor response with concomitant topical use of steroids or vitamin D was of significance (OR = 0.12, 95% CI 0.03–0.53, *p* = 0.005), but that with severe disease status was of borderline significance (OR = 0.29, 95% CI 0.08–1.04, *p* = 0.058).

### 3.9 Drug survival and reasons for drug discontinuation

**FIGURE 4:**
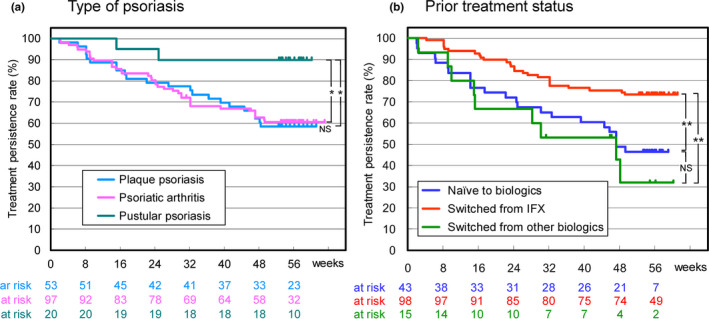
Kaplan–Meier plot of drug survival of CT‐P13 in patient groups classified by (a) type of psoriasis and (b) prior treatment status with biologics. Patients who discontinued treatment with CT‐P13 after a single dose were excluded from the plot. Statistical significance was analyzed by the log‐rank test. *p < 0.05, **p < 0.01. IFX, infliximab; NS, not significant.

The Kaplan–Meier curve of drug survival for patients with plaque psoriasis was almost the same as that for patients with psoriatic arthritis and drug survival rates after 1 year were approximately 60% (Figure 4a). Only two patients with pustular psoriasis discontinued CT‐P13 and the drug survival rate was significantly higher (90%) for pustular psoriasis.

Drug survival in patients switched from IFX was significantly higher compared with the other two patient groups and 74% of the patients were on CT‐P13 therapy at week 56 (Figure 4b). The drug survival rate in the naïve patients decreased to 47% at week 56. The drug persistence in patients switched from other biologics was lowest among the three groups, but not significantly different from that in naïve patients.

A total of 68 patients (41%) discontinued CT‐P13 treatment during the PMS period (Table 4). The most common reason for discontinuation was insufficient efficacy (*n* = 31, 19% of total patients) followed by AE (*n* = 15, 9%). CT‐P13 therapy was discontinued in seven and three patients due to patient decision and hospital policy, respectively. Among them, 14 patients were switched to the originator IFX, even though five patients had maintained PASI 0.

**TABLE 4:** Persistence of CT‐P13 treatment and reasons for drug discontinuation

Treatment persistence and reason for drug discontinuation	Total		Plaque psoriasis		Psoriatic arthritis		Pustular psoriasis		Psoriatic erythroderma		Naïve to biologics		Switched from IFX		Switched from other biologics	
	*n* = 165		*n* = 55		*n* = 104		*n* = 20		*n* = 2		*n* = 44		*n* = 105		*n* = 16	
Continuation of treatment over 1 year	97	(59%)	31	(56%)	58	(56%)	18	(90%)	1	(50%)	20	(45%)	72	(69%)	5	(31%)
Discontinuation of treatment	68	(41%)	24	(44%)	46	(44%)	2	(10%)	1	(50%)	24	(55%)	33	(31%)	11	(69%)
Remission	1	(1%)	0	(0%)	1	(1%)	0	(0%)	0	(0%)	0	(0%)	1	(1%)	0	(0%)
Insufficient efficacy	31	(19%)	12	(22%)	19	(18%)	1	(5%)	1	(50%)	10	(23%)	15	(14%)	6	(38%)
Adverse event	15	(9%)	5	(9%)	10	(10%)	1	(5%)	0	(0%)	5	(11%)	7	(7%)	3	(19%)
Patient decision	7	(4%)	3	(5%)	5	(5%)	0	(0%)	0	(0%)	2	(5%)	5	(5%)	0	(0%)
Hospital policy	3	(2%)	0	(0%)	3	(3%)	0	(0%)	0	(0%)	0	(0%)	3	(3%)	0	(0%)
Lost to follow‐up	11	(7%)	4	(7%)	8	(8%)	0	(0%)	0	(0%)	7	(16%)	2	(2%)	2	(13%)
Switching to IFX	14	(8%)	6	(11%)	9	(9%)	1	(5%)	0	(0%)	2	(5%)	12	(11%)	0	(0%)
Insufficient efficacy	10	(6%)	5	(9%)	5	(5%)	1	(5%)	0	(0%)	2	(5%)	8	(8%)	0	(0%)
Adverse event	1	(1%)	0	(0%)	1	(1%)	0	(0%)	0	(0%)	0	(0%)	1	(1%)	0	(0%)
Patient decision	3	(2%)	1	(2%)	3	(3%)	0	(0%)	0	(0%)	0	(0%)	3	(3%)	0	(0%)

*Note:* Data are expressed as number of patients (%).

Abbreviation: IFX, infliximab.

During the PMS period, the dose of CT‐P13 was augmented in 18 patients (11%) to overcome inadequate efficacy with the initial dose regimen. The dose was escalated (1.5 times or more) in 13 patients and the dosing interval was shortened (from 8 weeks to 6 weeks or less) in five patients. Of these 18 patients, nine (50%) succeeded to continue CT‐P13 therapy, even though they failed to respond to the initial regimen. The discontinuation rate due to insufficient efficacy was 44%, and ADR leading to drug discontinuation was observed only in one patient (6%) under the augmented dose regimen (Supporting Information Table S4).

## 4 DISCUSSION

This is the first report on the prospective PMS of a biosimilar in Japanese patients with psoriasis in real‐world clinical practice. In the safety analysis, the incidence of ADRs of CT‐P13 was 17.6%, which was lower than the previously reported incidence of 22.5% for IFX in PMS.^15^ The incidence of serious ADRs was also lower with CT‐P13 (1.2%) than IFX (6.9%), even though the observation period in this PMS was 1 year compared with 6 months in the former PMS. The lower ADR incidence of CT‐P13 in this PMS might be due to the higher proportion of patients with psoriatic arthritis among the four types of psoriasis (63% vs 32% in the PMS of IFX) and of patients previously treated with IFX before PMS entry (64% vs 4%). Japanese guidance for the use of biologics for psoriasis recommends starting treatment with TNF inhibitors from an early stage for patients with psoriatic arthritis to prevent the progression of joint destruction.^16^ In fact, patients with psoriatic arthritis in this PMS had the shortest disease duration (14.9 years) and the lowest proportion of severe disease (18%) among the four types of psoriasis. Among patients who switched from IFX, those who were sensitive to IFX were virtually excluded from CT‐P13 entry, and those with severe disease were rare (8%) although the disease duration was long (18.5 years).

The profile of ADRs observed with CT‐P13 was similar to that reported with IFX and no new safety signals were detected. The most common ADR was infusion reactions. Multivariable analysis revealed that previous use of other biologics and comorbidities were significant risk factors for infusion reactions. In addition, in univariate analysis, infusion reactions occurred significantly more frequently in patients with a history of drug allergy and with more severe disease status. Careful monitoring is required for patients with these clinical factors during the infusion.

Regarding the ADRs of interest, infection was reported in only one case with mild pneumonia and the incidence (0.6%) was much lower than that with IFX in PMS (5.1%). This is probably because proactive medication has been established since the clinical introduction of IFX. No cases of tuberculosis occurred. Risk of tuberculosis with anti‐TNF therapy is related to the local prevalence of latent tuberculosis,^23^ and the incidence of tuberculosis in Japan is still 12/100 000 in 2020.^24^ Therefore, patients should be closely monitored for the onset of tuberculosis prior to and after administration, as in this PMS. Interstitial pneumonia occurred in three patients: two patients recovered after discontinuing CT‐P13 without treatment, but one patient died. In the latter case, interstitial pneumonia developed after a single infusion of CT‐P13 following MTX, even though chest examinations prior to administration showed no abnormalities. His long smoking history and combination therapy with MTX were potential risk factors, but the causal relationship was unknown. Continuous careful attention should be paid to interstitial pneumonia.

Excellent efficacy of CT‐P13 for psoriasis was confirmed in this PMS. The response pattern varied among the three patient groups classified by prior treatment status with biologics: naïve patients to treatment with biologics, patients switched from the originator IFX, and patients switched from other biologics. In the first group of biologic‐naïve patients, CT‐P13 exhibited rapid improvement in all efficacy parameters evaluated: absolute PASI, PASI response, PGA, and DLQI. Comparing the effects of CT‐P13 for each type of psoriasis in naive patients, psoriatic arthritis exhibited a particularly high response, which might be due to the early use of CT‐P13, as mentioned in the safety analysis. CT‐P13 also improved psoriatic arthritis‐specific joint symptoms and ameliorated joint destruction, resulting in decreased DAS28‐CRP and a high remission rate.

Compared with efficacy data for IFX reported in a previous PMS,^15^ the PASI response in naïve patients was higher with CT‐P13 in the current PMS. The PASI 75 and PASI 90 response rates at week 30 by CT‐P13 were 85% and 65%, respectively, while those by IFX at 6 months were 60% and 42%, respectively. The difference in the efficacy may be a consequence of a paradigm shift in the treatment strategy toward using biologics in the early stages of psoriasis.^25^ In contrast to the higher response rate, the drug survival rate of CT‐P13 at 1 year in naïve patients was only 47%, which was generally lower than the previously reported drug survival rates of IFX in a real‐world setting. According to the first meta‐analysis of drug survival for biologics in real‐world treatment of psoriasis, the drug survival rate for IFX was 61% (95% CI 54%–67%) at 1 year in an analysis of 20 studies (published from 2011 to 2017) that included 2613 patients.^26^ A subsequent publication on Japanese psoriasis patients treated between 2010 and 2018 also reported that the drug survival rate for IFX at 1 year was 57% in 70 patients, including 56 naïve patients.^27^ The lower drug survival for CT‐P13 in the current PMS might be due to increased treatment options for recently approved biologics. An analysis of 1459 psoriasis patients treated with biologics from 2002 to 2018 in the Israeli healthcare database demonstrated that drug survival rate decreased by 1% per advancing calendar year, reflecting the availability of biologics.^28^ Aggressive treatment changes to achieve treatment goals according to the treat‐to‐target strategy may accelerate the decline in drug survival rates.^25^

No clinical difficulties were observed with the second group of patients switching from IFX to CT‐P13, consistent with results obtained for other inflammatory diseases such as rheumatoid arthritis and inflammatory bowel disease.^6^, ^8^, ^12^, ^14^ The disease activity of patients switched from IFX was generally controlled by previous treatment with the originator IFX. Subsequent administration of CT‐P13 further increased the percentage of patients with PGA <1 from 81% at baseline to 95% at week 56, and reduced the DLQI from 2.1 to 1.1. The incidence of ADRs was low (11.4%) and no serious ADRs were reported in this patient group. Consequently, the drug survival rate was as high as 74% after 1 year. The number of patients who discontinued treatment due to insufficient efficacy and AEs was only 15 (14%) and 7 (7%), respectively. Five patients (5%), however, decided to discontinue CT‐P13 treatment, even though three of them had maintained PASI 0. This could be explained by so‐called “nocebo effect” based on negative expectation for biosimilars.^29^ Furthermore, in this patient group, 12 patients discontinued CT‐P13 to switch back to IFX. Clinical data showing the equivalence of biosimilars with their originators, as described in this report, must be provided to overcome the nocebo effect and to show the cost‐effectiveness of biosimilar.

The third group of patients switched from other biologics had a long disease duration and complex treatment history with prior biologics. The proportion of patients who achieved absolute PASI <1 increased from 21% to 57% at week 30, similar to the response in naïve patients (12% to 55%). The percentage of patients with PGA <1 and DLQI, however, did not improve markedly as observed with naïve patients. ADRs were reported in three patients (18.8%) in this group, which was lower than in naïve patients (31.8%). Drug survival in patients switched from other biologics was lower than that in naïve patients after 1 year (32% vs 47%), but no significant difference was detected in the Kaplan–Meier analysis (Figure 4b). These results could be a practical basis for switching to CT‐P13 after treatment failure with other biologics.

We conducted multivariable logistic regression analysis to explore the predictive factors of the response to CT‐P13. We used absolute PASI <1 as the response criterion in the analysis instead of PASI response, which is commonly used, especially in clinical trials limited to moderate‐to‐severe psoriasis patients. It is not suitable for real‐world studies involving less severe patients because PASI response varies greatly depending on the PASI score before the start of treatment. The multivariable analysis revealed four independent predictors of response to CT‐P13. Male sex and psoriatic arthritis were significantly associated with a higher response rate, and BMI and use of topical steroids and/or vitamin D were associated with a poor response. These results generally corresponded to previous results of response to anti‐TNF agents, including IFX. De Simone et al.^30^ reported that male sex, psoriatic arthritis, and baseline PASI ≤15 were three significant factors associated with the PASI 75 response by multivariable analysis. Naldi et al.^31^ showed in an Italian nationwide cohort study that the proportion of patients who achieved PASI 75 was reduced with increased BMI. As well as efficacy, factor analyses were often performed on drug survival, and a recent meta‐analysis reported that female sex and obesity predicted drug discontinuation, and concomitant psoriatic arthritis predicted drug persistence.^32^ Although the underlying reasons for the relationship to efficacy are unclear, our results on the predictors of therapeutic response to CT‐P13 are of great importance in making treatment option decisions and reducing the economic burden for patients with psoriasis.

This study has several limitations. First, the number of patients analyzed in this PMS was limited. The prospective registration of all patients, however, might minimize patient selection bias. Second, the data collected for the PMS were inherently sparse compared with clinical trial data. In contrast, the collected real‐world information from a variety of patients with different background factors made it possible to extract safety risk factors and efficacy predictors. Finally, the PMS was not designed to compare with the originator and simple comparison of our data with historical data from a previous PMS of IFX could not be made due to changes in patient backgrounds with recent advances in psoriasis therapy. Instead, we could collect updated data on recent practical treatments and novel information on switching treatment with biologics.

In this prospective PMS, CT‐P13 exhibited sufficient efficacy in biologic‐naïve patients with psoriasis. In switching from IFX, CT‐P13 maintained the efficacy of previously administered IFX and drug survival was extended. Even in switched patients who failed to respond to other biologics, CT‐P13 showed similar efficacy to that observed in naïve patients. No new safety signal was added to those of the originator IFX. Therefore, CT‐P13 could be a cost‐effective alternative to IFX in the treatment of patients with psoriasis.

## CONFLICT OF INTEREST

A.M. has received research grants, consultancy fees, and/or speaker's fees from AbbVie, Amgen, Boehringer‐Ingelheim, Bristol‐Myers Squibb, Eisai, Eli Lilly Japan, Janssen Pharmaceutical, Kyowa Kirin, LEO Pharma, Maruho, Mitsubishi Tanabe Pharma, Nichi‐Iko Pharmaceutical, Nippon Kayaku, Novartis, Pfizer Japan, Sun Pharma Japan, Taiho Pharmaceutical, Torii Pharmaceutical, UCB Japan, and Ushio. K.N. was, and F.Y. is, an employee of Nippon Kayaku. K.Y. has received research grants, speaker's fees, and chair's fees from Nippon Kayaku. H.N. has received research grants, consulting fees, and/or speaker's fees from AbbVie, Amgen, Eli Lilly Japan, Janssen Pharmaceutical, Kyowa Kirin, Maruho, Novartis, Sun Pharma Japan, and UCB Japan. M.O. has received research grant, speaker's fees, and/or consultancy fees from AbbVie, Bristol‐Myers Squibb, Eisai, Eli Lilly Japan, Janssen Pharmaceutical, LEO Pharma, Maruho, Mitsubishi Tanabe Pharma, Nippon Kayaku, Novartis, Taiho Pharmaceutical, and Torii Pharmaceutical.

## Supporting information


Appendix S1
