# Supplementary material for: Safety, efficacy, and drug survival of the infliximab biosimilar CT‐P13 in post‐marketing surveillance of Japanese patients with psoriasis
Source: J Dermatol. 2022 Jul 7;49(10):957–69. doi: 10.1111/1346-8138.16508 (PMC9796256; doi:10.1111/1346-8138.16508)
Supplement: Supplementary file 1 — Appendix S1 [file JDE-49-957-s001.docx]

***Supporting Information***

**Title**

Safety, efficacy, and drug survival of the infliximab biosimilar CT-P13 in post marketing surveillance of Japanese patients with psoriasis

**Author**

Akimichi MORITA, Kiyohiro NISHIKAWA, Fumika YAMADA, Keiichi YAMANAKA, Hideki NAKAJIMA, Mamitaro OHTSUKI

**List of Figures & Tables**

Figure S1 Patient disposition.

Table S1 Incidence of ADRs and serious ADRs to CT-P13 by SOC.

Table S2 Univariate logistic regression analysis of baseline clinical factors associated with incidence of infusion reactions and other ADRs.

Figure S2 Efficacy of CT-P13 based on DAS28-CRP in patients with psoriatic arthritis.

Figure S3 Distribution of patients by PASI response in each body region evaluated 30/32 weeks after administration of CT-P13.

Table S3 Univariate logistic regression analysis of baseline clinical factors associated with response to CT-P13, that is achieving PASI score <1 between 14/16 and 30/32 weeks after CT-P13 administration.

Figure S4 Rate of response to CT-P13, that is achieving absolute PASI <1 according to increase in a) BMI and b) body weight.

Table S4 Conversion rate to augmentation therapy and persistence of CT-P13.


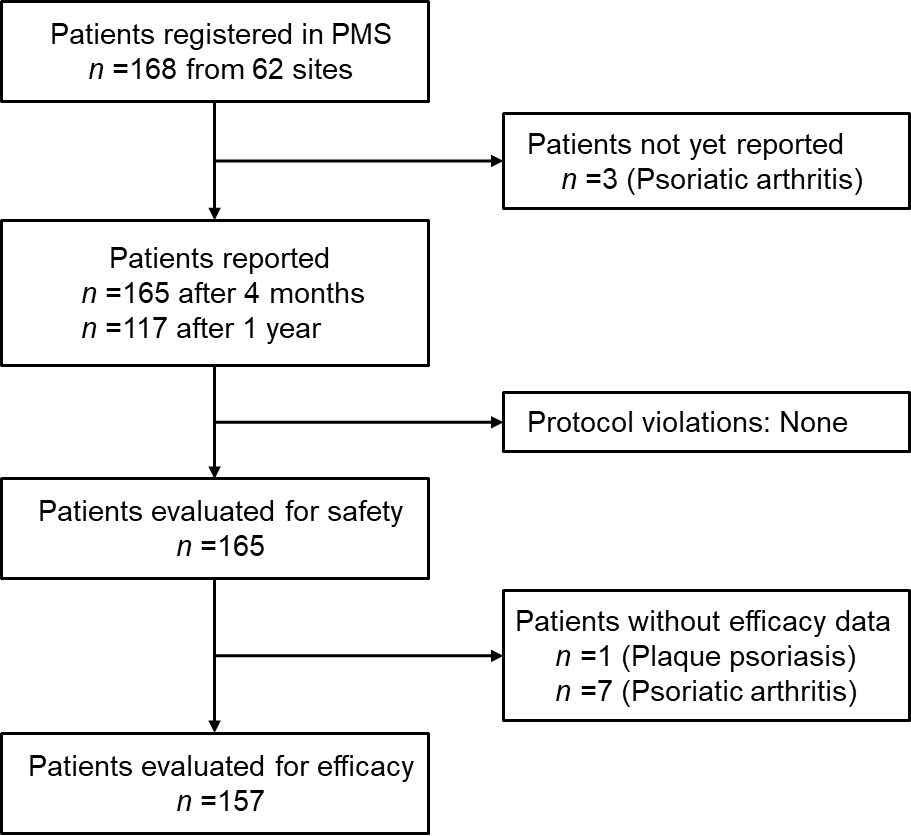


Figure S1 Patient disposition.

PMS, post-marketing surveillance.

Table S1 Incidence of ADRs and serious ADRs to CT-P13 by SOC

Data are expressed as number of patients with ADRs (%).

ADR, adverse drug reaction; SOC, System organ class.

Table S2 Univariate logistic regression analysis of baseline clinical factors associated with incidence of infusion reactions

and other ADRs

* P <0.05, ** P <0.01. ^†^ Disease severity is determined based on the rule of 10s (PASI score >10, BSA >10%, or DLQI >10).

ADRs, adverse drug reactions; BMI, body mass index; CI, confidence interval; IFX, infliximab; OR, odds ratio; MTX, methotrexate.


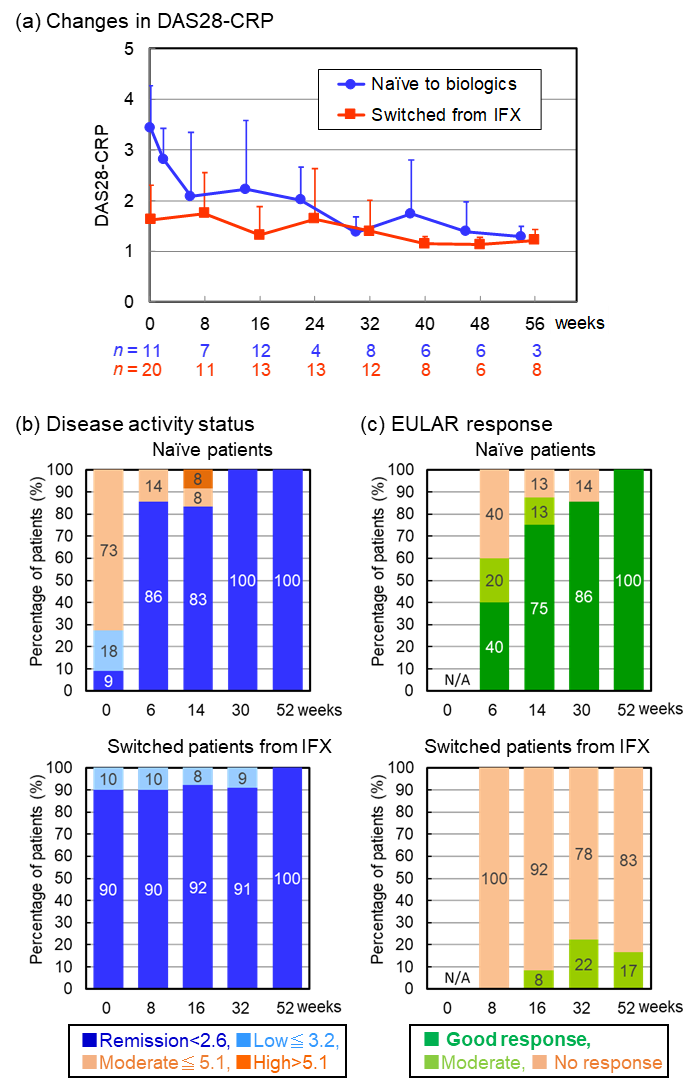


Figure S2. Efficacy of CT-P13 based on DAS28-CRP in patients with psoriatic arthritis. (a) Changes in DAS28-CRP (mean ± standard deviation) over time from baseline to week 56, (b) distribution of patients by disease activity status, and (c) distribution of patients who achieved a EULAR response.

DAS28-CRP, disease activity score in 28 joints with C-reactive protein; EULAR, European League Against Rheumatism; IFX, infliximab; N/A, not applicable.


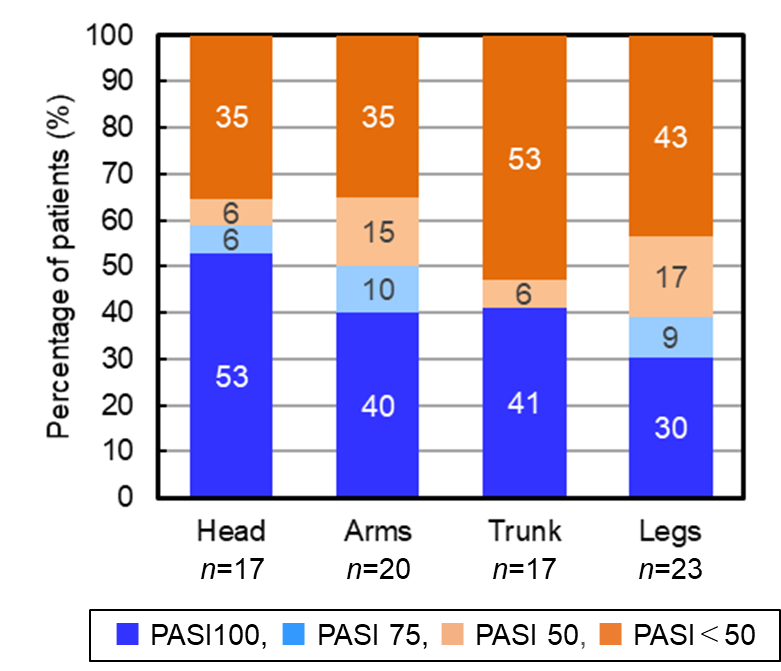


Figure S3. Distribution of patients by PASI response in each body region evaluated 30/32 weeks after administration of CT-P13.

PASI, Psoriasis Area and Severity Index.

Table S3 Univariate logistic regression analysis of baseline clinical factors associated with response to CT-P13, that is achieving absolute PASI <1 between 14/16 and 30/32 weeks after CT-P13 administration

* P <0.05, *** P <0.001. ^†^ Disease severity is determined based on the rule of 10s (PASI score >10, BSA >10%, or DLQI >10).

BMI, body mass index; CI, confidence interval; IFX, infliximab; OR, odds ratio; MTX, methotrexate.


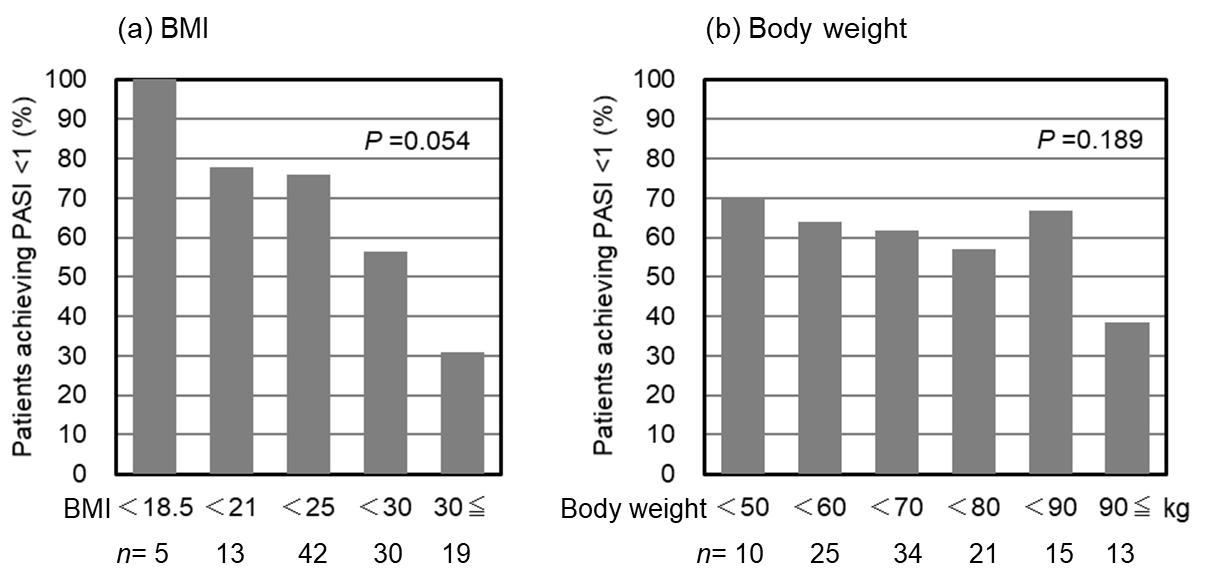


Figure S4. Rate of response to CT-P13, that is achieving absolute PASI <1 according to increase in a) BMI and b) body weight. Statistical significance was analyzed by the Cochran-Armitage test.

BMI, Body Mass Index; PASI, Psoriasis Area and Severity Index.

Table S4 Conversion rate to augmentation therapy and persistence of CT-P13.

^†^ Increased dosage over 1.5 times the initial dose. ^‡^ Shortened dosing interval from 8 weeks to 6 weeks or less. ^§^ Data are expressed as number of patients (% of total number of patients), ^¶^ Data are expressed as number of patients (% of patients with dose augmentation). IFX, infliximab.
